# Supplementary material for: Optimizing Treatment of Staphylococcus aureus Bloodstream Infections Following Rapid Molecular Diagnostic Testing and an Antimicrobial Stewardship Program Intervention
Source: Microbiol Spectr. 2023 Feb 15;11(2):e01648-22. doi: 10.1128/spectrum.01648-22 (PMC10101007; doi:10.1128/spectrum.01648-22)
Supplement: Supplemental file 1 — Supplemental material. Download spectrum.01648-22-s0001.pdf, PDF file, 0.2 MB [file spectrum.01648-22-s0001.pdf]

**TABLE S1** Patient demographics for study participants

| **Demographics** | **Control group (n=27)** | **Intervention group (n=29)** | ***P* value** |
|---|---|---|---|
| Age, mean (SD) | 68.7 (15.1) | 65.8 (14.8) | 0.469 |
| Sex, male, n (%) | 18 (66.7%) | 18 (62.1%) | 0.720 |
| Diabetes, n (%) | 10 (37.0%) | 10 (34.5%) | 0.842 |
| Chronic renal disease, n (%) | 5 (18.5%) | 10 (34.5%) | 0.178 |
| Admitting Service, n (%) | | | 0.523 |
| Hospitalist | 7 (25.9%) | 3 (10.3%) | |
| Medicine | 10 (37.0%) | 8 (27.6%) | |
| General surgery | 0 (0.0%) | 1 (3.4%) | |
| Orthopedics | 2 (7.4%) | 3 (10.3%) | |
| Cardiology | 1 (3.7%) | 1 (3.4%) | |
| Hematology | 1 (3.7%) | 1 (3.4%) | |
| Other services | 6 (22.2%) | 12 (41.4%) | |

Abbreviations: standard deviation (SD)

**TABLE S2** Standardized form used for feedback and guidance on methicillin-susceptible *Staphylococcus aureus* bloodstream infection management

<table>
<tr><td colspan="4">Technologist Name and Phone number to be reached at:</td></tr>
<tr><td colspan="4">Stewardship team member:</td></tr>
<tr><td colspan="4">Date and time of ASP phone call:</td></tr>
<tr><td colspan="4">Patient code:</td></tr>
<tr><td colspan="2">Age:</td><td colspan="2">Gender:</td></tr>
<tr><td colspan="2">Date of Admission:</td><td colspan="2">Unit:</td></tr>
<tr><td colspan="2">Admitting diagnosis:</td><td colspan="2">Receiving Physician name and service:</td></tr>
<tr><td colspan="4">Number of Blood culture sent :</td></tr>
<tr><td colspan="4">Date Blood culture collected: time</td></tr>
<tr><td colspan="4">Date blood culture flags positive: time</td></tr>
<tr><td>Gram stain results:</td><td>Organism ID by MALDI (direct blood)</td><td>Organism ID by MALDI (plate growth)</td><td>Organism ID by GeneXpert</td></tr>
<tr><td colspan="4">Other positive cultures (ID / site):</td></tr>
<tr><td colspan="4">Potential source of bacteremia:</td></tr>
<tr><td colspan="2">Current antibiotics:<br>Dose<br>Start date</td><td colspan="2">Allergies:<br>Renal function (CrCL):</td></tr>
<tr><td colspan="4">Recommendations</td></tr>
<tr><td colspan="4">• Dosage for patient with normal renal function,<br>  • Cefazolin 2 g IV q8h (adjust for renal function)<br>  • Cloxacillin 2 g IV q4h<br>  • Vancomycin 25 mg/kg loading dose (then 15 mg/kg IV q12h), draw trough level pre 4th dose<br>  • Daptomycin 6mg/kg IV q 24 hours<br>• Repeat blood cultures q48hours <u>until negative</u> to demonstrate sterilization<br>• ECHO (TEE preferred)<br>• Consider ID consult<br>• https://library.nshealth.ca/AMS/Handbook#s-lg-box-15905948</td></tr>
<tr><td colspan="4">Notes</td></tr>
</table>

**TABLE S3** Exclusion criteria for intervention and control groups

| **Control group** | | **Intervention group** | |
|---|---|---|---|
| Isolates screened for inclusion | 34 | Isolates with Xpert performed | 44 |
| Reasons for exclusion: | | Reasons for exclusion: | |
| Methicillin-resistant *Staphylococcus aureus* | 1 | Methicillin-resistant *Staphylococcus aureus* | 3 |
| Palliative | 3 | Palliative | 3 |
| Polymicrobial infection | 1 | Polymicrobial infection | 2 |
| Left against medical advice | 1 | Alternative definitive beta-lactam therapy | 2 |
| Transferred to external hospital | 1 | Transferred to external hospital | 2 |
| | | Ongoing hospitalization | 2 |
| | | Not admitted | 1 |
| **Included in final analysis** | **27** | **Included in final analysis** | **29** |

**TABLE S4** Hazard ratios for the primary and secondary outcomes, intervention group relative to control group

| | **HR**** | **95% CI** | ***P* value** |
|---|---|---|---|
| TTOT | 1.77 | (1.02, 3.09) | 0.043 |
| TTAT | 1.47 | (0.85, 2.54) | 0.171 |
| Duration of vancomycin use* | 2.32 | (0.78, 6.89) | 0.130 |

*Based on the n=17 patients who had continuous vancomycin use prior to optimal therapy.

** the proportional hazards assumption is valid for each outcome according to the Kolmogorov-type supremum test ($p>0.05$).

Abbreviations: confidence intervals (CI); hazard ratio (HR); time to appropriate therapy (TTAT); time to optimal therapy (TTOT).
